# Type 2 Diabetes Mellitus and Alzheimer’s Disease: Role of Insulin Signalling and Therapeutic Implications

**DOI:** 10.3390/ijms19113306

**Published:** 2018-10-24

**Authors:** Andrea Tumminia, Federica Vinciguerra, Miriam Parisi, Lucia Frittitta

**Affiliations:** Endocrinology, Department of Clinical and Experimental Medicine, University of Catania, Diabetes, Obesity and Dietetic Center, Garibaldi Hospital, Via Palermo n° 636, 95122 Catania, Italy; andreatumminia@libero.it (A.T.); vinciguerrafederica@gmail.com (F.V.); mrmparisi@gmail.com (M.P.)

**Keywords:** insulin receptor, insulin signalling, neurodegenerative disorders, Alzheimer’s disease, type 2 diabetes mellitus

## Abstract

In the last two decades, numerous in vitro studies demonstrated that insulin receptors and theirs downstream pathways are widely distributed throughout the brain. This evidence has proven that; at variance with previous believes; insulin/insulin-like-growth-factor (IGF) signalling plays a crucial role in the regulation of different central nervous system (CNS) tasks. The most important of these functions include: synaptic formation; neuronal plasticity; learning; memory; neuronal stem cell activation; neurite growth and repair. Therefore; dysfunction at different levels of insulin signalling and metabolism can contribute to the development of a number of brain disorders. Growing evidences demonstrate a close relationship between Type 2 Diabetes Mellitus (T2DM) and neurodegenerative disorders such as Alzheimer’s disease. They, in fact, share many pathophysiological characteristics comprising impaired insulin sensitivity, amyloid β accumulation, tau hyper-phosphorylation, brain vasculopathy, inflammation and oxidative stress. In this article, we will review the clinical and experimental evidences linking insulin resistance, T2DM and neurodegeneration, with the objective to specifically focus on insulin signalling-related mechanisms. We will also evaluate the pharmacological strategies targeting T2DM as potential therapeutic tools in patients with cognitive impairment.

## 1. Introduction

The brain was once thought to be an insulin-insensitive organ. However, it is now widely recognized that insulin plays an important role in neuronal survival and brain function. Insulin action is, in fact, required for neuronal synaptic plasticity and facilitates learning and memory [1]. It has also been shown that insulin promotes dendritic spine and synapse formation, neuronal stem cell activation, neurite growth and repair and neuroprotection [2]. Therefore, alterations in insulin metabolism and signalling in the Central Nervous System (CNS) can contribute to the development of many brain disorders.

Over the past 20 years, many studies have shown an association between neurodegenerative disorders such as Alzheimer’s Disease (AD) and impaired insulin signalling in CNS [3,4], suggesting that reduced insulin action and insulin resistance might play an important role, through different mechanisms, in the pathogenesis of these brain disorders.

Following, we will review the main pathophysiological connection between AD and T2DM, highlighting the role of dysfunctional insulin transduction pathway in the determinism of neurodegeneration. We will focus on the role of insulin signalling in the crucial hallmarks of AD-related damage: deposition of neuritic plaques, formation of intracellular neurofibrillary tangles (NFTs), vasculopathy and inflammation-related damage.

We will also evaluate the pharmacological treatments targeting type 2 diabetes (T2DM) as potential therapeutics for preventing neurodegeneration and cognitive decline.

## 2. Overview of Insulin Signalling

Insulin and Insulin-Like-Growth-Factor (IGF)-1 regulate several biological processes through the binding and activation of two closely related tyrosine kinase receptors, the Insulin Receptor (IR) and the IGF-1 receptor (IGF-1R) [5]. Several studies have demonstrated that IR and IGF-1R, as well as their common downstream pathways, are widely distributed within the brain and, more importantly, these pathways function as regulators of neurogenesis, brain function and whole-body energy balance and metabolism [3]. The highest concentration of IR is in the hypothalamus, hippocampus, in the olfactory bulb, cerebellum, amygdala and cerebral cortex [6]. The wide spectrum of insulin receptors’ location within the CNS indicates multi-functionality of insulin [3]. 

Insulin is a large peptide hormone that cannot passively pass through the blood-brain barrier (BBB) but it is still found in the cerebrospinal fluid (CSF). The origin of “brain” insulin is controversial. One possibility is that plasma insulin is able to cross the BBB via a saturable transport process, possibly the IR on vascular endothelium. Supporting this hypothesis is the evidence that insulin levels in the CSF are lower (about 25% less) of those circulating in blood and its concentrations increase after meals or with peripheral insulin infusion [7]. Another possibility is the evidence that there are regions of the brain, such as the hypothalamus, that lack an effective barrier, allowing insulin access to CNS [8]. A third hypothesis suggests that insulin is synthesized in the brain regions but this possibility requires further studies [3,9].

After reaching the CNS insulin binds to the IR, which belongs to the family of tyrosine kinase receptors. Interestingly, IR subunits found in the brain have different structure to those of the periphery and the main difference is a lower molecular weight of brain IR subunits probably due to different glycosylation [10]. Moreover, the brain expresses predominantly the A isoform (-exon 11) of the IR, which has a higher affinity for IGF-2, in contrast to the peripheral tissues which express predominantly the B isoform (+exon 11) [11,12].

Insulin is suggested to have neuroprotective properties and to exert neurotrophic effects on CNS neurons [13]. Moreover, it could positively influence emotion and higher cognitive processes including attention, executive functioning, learning and memory [14].

**Figure 1:**
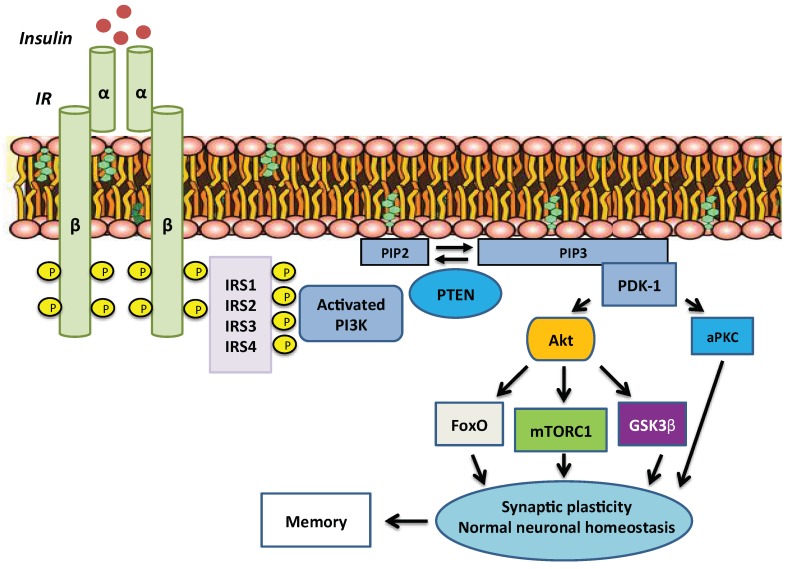
Insulin signalling pathway. After insulin binding to the insulin receptor, autophosphorylation, which is essential for its activation, occurs. Then, the activated insulin receptor phosphorylates IRS proteins. IRSs activate PI3K, which catalyses the addition of a phosphate group to the membrane lipid PIP2, thereby converting it to PIP3. PTEN can convert PIP3 back to PIP2. Membrane-bound PIP3 recruits and activates PDK-1, which phosphorylates and activates Akt and atypical PKCs. Akt mediates most of insulin’s metabolic effects and in brain synaptic plasticity, neuronal homeostasis and memory. Abbreviations: IRS (insulin receptor substrate), PI3K (phosphatidylinositol 3 kinase), PIP2 (phosphatidylinositol 4,5-bisphosphate), PIP3 (phosphatidylinositol 3,4,5-trisphosphate), PTEN (phosphatase and tensin homolog), PDK-1 (phosphoinositide-dependent protein kinase-1), PKC (protein kinase c), Akt (protein kinase b), mTORC1 (mammalian target of rapamycin complex 1), GSK3β (glycogen synthase kinase 3β), FoxO (forkhead box O).

After insulin binding to the IR, auto-phosphorylation of the receptor occurs and the activated IR phosphorylates a cascade of IR substrate proteins (Figure 1) [5]. Among the IR substrates (IRSs), IRS-2 mRNA in the brain is most abundant compared with IRS-1; IRS-4, which is mainly expressed in embryonic development, is also expressed in the brains of adult mice, especially the hypothalamus [3,15]. At the whole-body level, IRS-1 appears to be critical for growth and IRS-1-null mice result in an increased brain-to-body ratio [16]. On the other hand, disruption of IRS-2 gene reduces neuronal proliferation during development by 50% and as a consequence IRS-2-null mice exhibit a reduced brain-to-body ratio [17]. In addition, during aging, neurofibrillary tangles containing phosphorylated tau accumulated in the hippocampus of IRS-2 knockout mice, suggesting that IRS-2 signalling is neuroprotective [17]. Despite this, IRS-2-null mice are long lived [18], consistent with a role of central insulin/IGF signalling in control of life span in mammals. IRS-4 may synergistically cooperate with IRS-2 in the hypothalamus to control food intake, energy expenditure and glucose metabolism [19]. 

The specific inactivation of IR in the brain (i.e., neuron-specific IR knockout—NIRKO—mice), showed that lack of IR in brain determine an altered metabolic phenotypes, including diet-sensitive obesity, increased body fat and insulin resistance while did not affect brain size and development, suggesting that IR signalling in the CNS plays an important role in regulation of energy disposal and fuel metabolism [3,20]. Moreover, insulin resistance in the brain exists as a distinct phenomenon, independent of peripheral insulin resistance and glucose intolerance [21,22]. It implies that diminished responsiveness to insulin in the brain has different consequences than in peripheral tissues. Recent published data showed that peripheral insulin and glucose tolerance were comparable between aged wild type and APP/PS1 mice (a model of AD), while levels of serine phosphorylated IRS-1 were increased in the brain of APP/PS1 mice [23]. This provides some support for the suggestion that insulin resistance in brain may indeed exist as a distinct phenomenon, separate from insulin signalling in the periphery and one that can distinguish AD-related neurodegeneration from normal aging. 

One of the major downstream pathways of IRS proteins is the PI3K/Akt cascade. This, in turn, targets multiple downstream pathways, including mTORC1, glycogen synthase kinase 3β (GSK-3β) and the FoxO family of transcription factors (Figure 1) [24]. Many of these pathways have been shown to play pivotal roles in normal brain function.

## 3. T2DM and Neurodegeneration: The Role of Impaired Insulin Signalling, Insulin Resistance and Hyperinsulinemia

**Figure 2:**
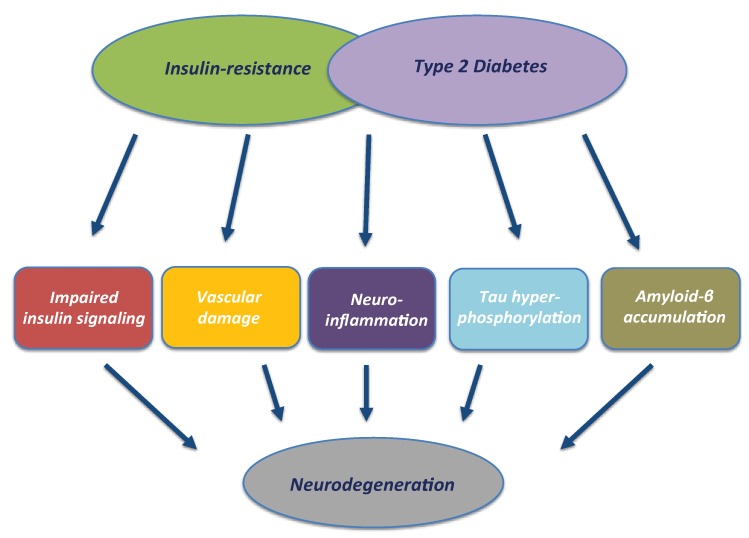
Contribution of T2DM and insulin resistance to neurodegeneration. Metabolic changes resulting from T2DM and insulin resistance can impact on the brain, resulting in synaptic dysfunction and promoting triggers/drivers of neurodegeneration: impaired neuronal insulin signalling, vascular damage, neuroinflammation, tau phosphorylation and Aβ accumulation.

T2DM is a complex, age-related chronic disease and the increasing prevalence is also of great public concern. Currently, more than four hundred million people have diabetes mellitus worldwide and this number is expected to increase dramatically by the next thirty years [25]. T2DM, characterized by cellular insulin resistance and chronic inflammation, causes accelerated aging [26] and leads to premature morbidity and mortality. The effects of T2DM on the brain are now well recognized: it is known to be a major risk factor for cognitive decline and dementia. In fact, T2DM increases the long-term risk of dementia by nearly 2-fold and one in ten cases of dementia in the world population may be attributable to the effects of T2DM [4]. The reciprocal link in the prevalence of these chronic disorders is due to the fact that diabetes and dementia share several important features leading to brain damage, the most important of which are the impaired insulin sensitivity, Aβ accumulation, tau hyper-phosphorylation, vascular damage and inflammation (Figure 2). The systematic examination of all these connections is not the objective of the present review. Instead we will focus on the role of insulin signalling in the crucial hallmarks of AD-related damage.

Alzheimer’s disease (AD) is a chronic neurodegenerative disease that usually starts slowly and worsens over time. It is the cause of 60–70% of cases of dementia [27]. The most common early symptom is difficulty in remembering recent events (short-term memory loss). As the disease advances, symptoms can include problems with language, disorientation, mood swings, loss of motivation, not managing self-care and behavioural issues [28]. This progressive neurodegenerative disease is characterized by the accumulation in the brain of extracellular neuritic plaques and fibrils (primarily consisting of aggregated of Amyloid β—Aβ—peptides), intracellular neurofibrillary tangles (accumulation of hyper-phosphorylated protein tau—NFTs), microglial infiltration, brain atrophy and widespread synaptic and neuronal loss. Enhanced neuroinflammation is also consistently observed in AD [29] and evidence suggests that early hyperactivity of pro-inflammatory pathways in the brain precedes the development of plaques and tangles in AD [30].

Several pathophysiological links have been demonstrated between AD and metabolic disorders such as T2DM, obesity and metabolic syndrome [31,32,33]. In contrast to a small subset of AD cases (~3%) attributable to inherited genetic causes, the pathogenesis and aetiology of sporadic, late onset AD (LOAD) are multifactorial, involving genetic and life-style risk factors [34]. Failure to consider these additional metabolic aspects of AD limits opportunity to fully understand the nature of disease and therapeutically target its underlying basis.

The recognition of T2DM as a major risk factor for dementia, particularly AD, has driven research to understand the underlying mechanisms linking these two age-related chronic disorders. Metabolic disturbances associated with the diabetic phenotype (i.e., hyperglycaemia, hyperinsulinemia, hypercholesterolemia) are known to be associated with brain atrophy and the pathological features of AD [3]. Whether insulin resistance is a cause or consequence of AD is still not clear; however, insulin action has been shown to play a role in several important parts of the progressive pathogenesis of AD (Figure 2).

Lower brain glucose uptake and impaired glucose metabolism are associated with AD [35]. Moreover, many findings support the concept that insulin-signalling dysregulation may be a key early contributing factor in AD pathogenesis. Several mechanisms can explain the correlation between AD and insulin signalling defects. In fact, some studies showed that the expression and activation of IR, IGF-1R and IRS-1 proteins is reduced in the brain of AD patients compared to healthy control brains [36]. Moreover, some authors demonstrated that neocortical levels of insulin and binding to insulin receptors are reduced in AD brains [37]. Finally, a lower insulin concentration in the CSF in spite of higher plasma insulin concentration [38], suggests reduced insulin action in the CNS.

## 4. Insulin Signalling and Metabolism and Aβ Deposition

The amyloid plaques found in the brains of patients with AD are mainly composed of Aβ, a peptide derived from a larger molecule that is known as the amyloid precursor protein (APP). APP is normally cleaved within its extracellular domain by the enzyme called “α-secretase”. Damaging Aβ originate from proteolysis of the APP by the sequential enzymatic actions of beta-site amyloid precursor protein–cleaving enzyme 1 (BACE-1), a β-secretase and γ-secretase. An imbalance between production, clearance and aggregation of peptides, causes Aβ to accumulate and this excess may be the initiating factor in AD [39].

Several evidences suggested a link between energy metabolism defects to functional alterations associated with pathogenesis of AD [40]. Inhibition of energy metabolism can alter APP processing and induce amyloidogenic products [41]. The relationship between insulin and Aβ metabolism is recently receiving increasing attention and a direct link between the Aβ metabolism and insulin pathways has been described in neuronal cell lines [42].

It is known that small oligomers of Aβ contribute to the synapto-toxicity and downstream events that lead to the neurodegenerative processes in AD [43,44]. As part of these neurodegenerative processes, Aβ oligomers appear to have detrimental effects on insulin signalling by inhibiting auto-phosphorylation of the receptor [45] and to markedly reduce IR levels and activity at the cell surface of the dendrites of hippocampal neurons [46]. IR plays a key role in the important neurological processes including learning and memory and tau phosphorylation. Thus, Aβ oligomer-induced loss of membrane IRs might represent important early mechanism underlying memory impairment and other pathological features of AD.

Aβ oligomers have also been found to determine aberrant activation of the TNFα/JNK and IRS-1 inhibition in both in vitro and in vivo models [21,47]. Furthermore, Aβ oligomers also have effects on signalling downstream from IRS-1 and PI3K, where they can activate serine phosphorylation of Akt and promote inflammatory processes [48]. On the other hand, insulin resistance is known to accelerate Aβ production promoting its accumulation. When insulin resistance is induced in transgenic AD mice or in diabetic obese mice through feeding a high fat diet, the mice exhibit increased brain Aβ levels and increases in levels of the key enzymes that generate Aβ (like γ-secretase) [49]. Finally, insulin and Aβ are both substrates of insulin-degrading enzyme and it has been suggested that hyperinsulinemia inhibits the degradation of Aβ by competitively blocking insulin-degrading enzyme [50].

## 5. Impairment of Insulin Signalling and Tau Hyper-Phosphorylation

Deficiencies in insulin signalling could also exacerbate neurodegeneration by enhancing the phosphorylation of tau protein. Tau is a neuronal microtubule-associated protein found in axons. It plays an important role in assembly and stability of microtubules as well as in vesicle transport in neurons. In AD, the hyper-phosphorylation of the microtubule-associated protein tau to form NFTs is an important pathological hallmark and smaller aggregates of hyper-phosphorylated tau are considered to contribute to neuronal dysfunction and degeneration [51]. It has been shown that insulin and IGF-1 regulate tau phosphorylation through the inhibition of GSK-3β in cultural neurons [52]. GSK-3β represents a key kinase that phosphorylates tau. Deficiencies or impairments in brain insulin signalling lead to a reduction in Akt activity resulting in an increase in GSK-3β activity. This phenomenon produces the hyper-phosphorylation of tau and, therefore, the formation of tau fibrils [53]. Moreover, peripheral hyperinsulinemia promotes tau phosphorylation in vivo [54]. It has been demonstrated that when IGF-1 and IRS-2 gene are deleted, tau phosphorylation is dramatically increased in IGF-1 and IRS-2 knockout mice [17,55]. Indeed, IGF-1 genetic deletion specifically increases tau phosphorylation at two specific GSK-3β targeted sites [55]. These results suggest that insulin and IGF-1 signalling normally prevents tau hyperphosphorylation in the brain. Given that T2DM is characterized by insulin resistance, hyperinsulinemia and impaired insulin signalling, it is not surprising that an increased GSK-3β activity in T2DM might lead to an elevation of Aβ production [56] and increased tau phosphorylation [57].

Insulin can also regulate tau expression and reduced insulin signalling can result in impaired tau gene expression [48] leading to reduced levels of normal soluble tau whilst hyper-phosphorylated tau accumulates, exacerbating neuronal cytoskeletal collapse, neurite retraction and impairments in synapse formation. Moreover, it has been demonstrated that the AD-associated reduction in tau mRNA expression is correlated to the impaired insulin and IGF-1 signalling observed in the same AD samples [58] demonstrating the strong connection between the two mechanisms.

## 6. Insulin Resistance, Brain Vasculopathy and Neuroinflammation

Neurodegenerative disorders and T2DM are both characterized by vascular damage, reduced cerebral flow and aberrant inflammatory response [59].

It has been demonstrated that AD patients have a decreased regional cerebral blood flow that could result in decrease brain supply of oxygen, glucose and nutrients [59,60]. This phenomenon is partially mediated by an impaired insulin transduction pathway. Insulin signalling, in fact, is involved in the regulation of vasodilation and vasoconstriction [61]. IR activation mediates vasodilation through PI3K/Akt pathway. It stimulates endothelial nitric oxide synthase (eNOS) resulting in the production of nitric oxide (NO) and vascular relaxation [61]. In insulin resistant state, there is a specific impairment in the vasodilatory PI3K pathway, resulting in decreased NO production and, therefore, leading to vasoconstriction. The resultant decrease in nutrient availability to the brain determines an increase of oxidative stress and reactive oxygen species (ROS) production and, consequently, an increased inflammatory response. Released pro-inflammatory cytokines and macrophage recruitment instigates the onset of atherosclerosis, ultimately leading to macrovascular complications [59].

It is well recognized that inflammatory processes form a major part of the pathogenesis of T2DM as well as neurodegenerative diseases. Indeed, there is mounting evidence that such induced chronic inflammation is an important early stage of AD pathogenesis [62,63].

Hyperinsulinemia has been shown to promote inflammatory responses in the CNS [64]. Increased peripheral insulin levels have been found to lead to increased cerebral levels of pro-inflammatory cytokines such as interleukin-1β (IL-1β), interleukin-6 (IL-6) and tumour necrosis factor-α (TNF-α), all of which have been reported to be elevated in AD and have been located in amyloid plaques and their related glial cells in AD patients [65].

In peripheral insulin resistance, the production of inflammatory cytokines and activation of inflammatory stress signalling, can lead to serine phosphorylation of IRS-1 by kinases, inhibitor of kappa B kinase (IKK), c-Jun N-terminal Kinase (JNK) and extracellular signal-regulated kinase 2 (ERK2), which in turn interfere with IR-mediated signalling, blocking the intracellular actions of insulin [66]. A similar mechanism has been suggested to occur in the brain, where Aβ oligomers can activate microglia, resulting in the secretion of pro-inflammatory cytokines, which bind to their respective receptors, activating one or more of the IRS-1 serine kinases [47] and in turn phosphorylating IRS at multiple sites. Elevated levels of vascular pro-inflammatory cytokines, observed in both T2DM and AD, can also impact on brain insulin signalling. When cerebral vascular tissue is damaged, cytokines can, in fact, cross the BBB and can activate phosphorylation of IRS-1 [67,68].

Vascular inflammation can also be mediated by the receptor for advanced glycation end products (RAGE). RAGE is expressed in neuronal cells, microglia astrocytes and in brain endothelial cells and levels are increased in both AD and T2DM. Increased RAGE levels have been proposed as a possible mechanism for vascular dysfunction in both T2DM and AD [69] and the interactions between disturbed cerebral glucose metabolism, oxidative stress and the accumulation of AGE’s are important in the vicious cycle that contributes to AD progression [70]. RAGE is an avenue for receptor mediated transport of Aβ across the BBB from the periphery to the brain [71], inducing cerebrovascular dysfunction resulting in neurovascular stress, production of TNF-α and IL-6, contributing to synapto-toxicity and neurodegeneration [69].

## 7. Therapeutic Implications

As T2DM shares several characteristics with neurodegenerative disorders, as previously discussed, it has been suggested that several drugs used in the treatment of T2DM could have potential benefits in AD (Table 1).

**Metformin**

Metformin is a biguanide that reduces insulin-mediated hepatic glucose production, increases insulin sensitivity and represents first-line treatment for T2DM. It rapidly crosses the BBB, distributes into several brain regions [105] and, by the activation of the AMPK pathway, seems to have a neuroprotective effect on human neural stem cells, restoring mitochondrial functions and attenuating AGEs effects [72,73].

Data on effects of metformin in neurodegenerative disorders are contrasting. In vitro studies reported the ability of metformin to reduce phosphorylation of tau, in neuronal cell lines [74] or in murine primary neurons from wild type and human tau transgenic mice [75]. In vivo studies have shown that in obese leptin resistant mice, metformin attenuated cognitive impairment and AD-like pathology [76]. In contrast, a study on cell culture showed that metformin increases the generation of amyloid beta protein [77].

In humans, observational studies show reduction in mild cognitive impairment (MCI) [78] and dementia [85,106] among participants with diabetes taking metformin when compared with no medication or other glucose-lowering agents. Long-term treatment with metformin seems to decrease the risk of cognitive decline in diabetic patients [78] and improve depressive and cognitive performance, changing the glucose metabolism, in depressed patients [79]. A recent pilot clinical trial in patients with MCI, randomly assigned to receive metformin or placebo for 12 months, showed that metformin improved cognition in individuals without diabetes [107].

A clinical study examining the effect of different diabetes treatments on cognitive function showed that diabetic patients who used metformin alone had a better cognitive function for the domain of verbal learning, working memory and executive function compared to participants on other forms of diabetic treatment [80].

On the other hand, an increased risk of cognitive impairment and developing AD was demonstrated with the use of metformin in a study conducted on AD patients [81]. This phenomenon was partially mediated by the metformin-induced vitamin B12 deficiency; in fact, it became weaker after adjusting for serum vitamin B12 levels. Nevertheless, the results of an analysis of cognitive function, measured 8–10 years after therapy with metformin in the Diabetes Prevention Program Outcomes Study (DPPOS) [108], did not show any negative impact from long-term metformin use.

Upcoming randomized clinical trials will evaluate whether metformin can prevent cognitive decline or improve cognitive functions in humans [109].

**Sulfonylureas**

Sulfonylureas are antidiabetic drugs that stimulate insulin release by blocking ATP sensitive potassium channels on pancreatic beta cells. In vitro, glimepiride protects neurons against beta- amyloid-induced synapse degeneration [82]. In streptozotocin-induced diabetic rats, gliclazide was able to exert an antioxidant effect in the brain [83]. Also, glibenclamide decreases depression and anxiety in a rat model of AD [84].

An 8 years clinical prospective study conducted on diabetic patients showed that the combination of sulfonylureas and metformin reduced the risk of dementia [85], however, another case control study demonstrated that long term use of sulfonylureas does not modify risk of dementia [110].

More extensive studies are needed to verify the potential therapeutic role of this class of drugs.

**Thiazolidinediones (Glitazones)**

Thiazolidinediones (TZDs), pioglitazone and rosiglitazone, are potent and selective receptor agonist of peroxisome proliferator–activated receptor-γ (PPAR-γ) nuclear receptors that improve insulin sensitivity in muscle, adipose and hepatic tissue; reducing systemic insulin resistance. TZDs may have a role on improvement of neuronal function and memory formation. These drugs showed a neuroprotective effects in AD related to inhibition of inflammatory gene expression and alteration of amyloid beta generation and deposition [86].

Pioglitazone, the only TZD actually available in commerce, is able to enter the brain, suppress glial activation and reduces AD-related pathologies [87].

In mouse models of AD, pioglitazone administered for 4 months enhances Akt signalling, attenuates spatial learning impairment and tau hyperphosphorylation [88]. Moreover, used in combination therapy with leptin reduces spatial memory deficits and brain amyloid levels [89].

In human, a pilot study showed improvement of cognition and regional cerebral blood flow in the parietal lobe of subjects with T2DM treated with pioglitazone for 6 months [90]. However, an 18-months trial in nondiabetic patients with AD, designed to evaluate the safety of pioglitazone, showed as secondary outcomes no treatment effects in cognition [111].

A meta-analysis on effect of PPAR gamma agonist in AD patients demonstrated that only pioglitazone may provide an improvement in the early stages and in mild-to-moderate AD [91,112].

A phase 3 clinical trial on the efficacy of pioglitazone in patients with mild cognitive impairment is ongoing, also using an algorithm to evaluate genetic-based biomarkers for preclinical diagnosis such as APOE status and genotypes. (Clinical trial identifier: NCT01931566). Data will be available in 2019. 

**Glucagon-Like Peptide-1 (GLP-1) Receptor Agonists**

Another class of antidiabetic drugs is that of the GLP-1 receptor agonists. GLP-1 is an incretin peptide secreted by the intestine that enhance glucose-dependent insulin secretion and inhibits glucagon secretion for maintaining glucose homeostasis. GLP-1 also has trophic properties as stimulation of β-cell neogenesis, growth and differentiation, inhibition of β-cell apoptosis and enhancement of cell survival [113,114].

GLP-1 and most of analogues cross over the BBB and GLP-1 receptor is expressed throughout the brain, in the frontal cortex, hypothalamus, thalamus, hippocampus, cerebellum and substantia nigra [115]. GLP-1 has a neuroprotective role: in the brain of AD mouse models, it seems to reduce apoptosis, protect neurons from oxidative stress and synapses from the harmful effects of reduced synaptic plasticity in the hippocampus induced by beta-amyloid [92]. Native GLP-1 has short half-life because is easily degraded by dipeptidyl peptidase-4 (DPP-4). Several GLP-1 analogues, more stable and more slowly degraded than GLP-1 native, have been developed; among these, exenatide, liraglutide and lixisenatide cross the BBB and, independently from their effects on glucose control, influence cellular pathways of neuronal protection, mitochondrial function, apoptosis and oxidative stress [93]. For their neuroprotective effects, GLP-1 analogues have been investigated as potential treatment of AD and other neurodegenerative disorders. In studies on AD mouse models, GLP-1 analogues reduced neuronal tau hyperphosphorylation, prevented synaptic loss, improved motor function, enhanced synaptic plasticity, attenuated memory and learning deficits and diminished beta amyloid plaque load in the brain [94,95]. The neuroprotective effects of liraglutide seem to be mediated through the PI3K-Akt signalling pathway [116] while those of lixisenatide were attributed to induced Akt and MEK signalling pathways [117].

A pilot clinical trial on humans showed that 6 months of liraglutide treatment in AD patients with long-standing disease prevents the decline of brain glucose metabolism expected as a sign of disease progression; in this study, however, no differences were found in beta- amyloid deposition or in cognition [96]. Other phase II trials evaluating GLP-1 analogues in patients with AD are ongoing.

Certainly, GLP-1 analogues have the advantage of not affecting blood sugar levels in nondiabetic people and so may represent a potential safe treatment for AD or other neurodegenerative conditions also in non-diabetic patients.

**Dipeptidyl Peptidase-4 Inhibitors (DPP-4i)**

DPP-4i (gliptins) are antidiabetic drugs that, inhibiting DPP-4, proteolytic enzymes responsible for degradation of GLP-1, extend its plasma half-life, stabilizing its level and inducing a functional enhancement of its antidiabetic effect. DPP-4 inhibitors have shown neuroprotective effects that could be partially mediated through the effects of GLP-1 in the brain.

In AD animal models, DPP-4i inhibitors treatment (saxagliptin, vildagliptin, sitagliptin) decreases tau phosphorylation, amyloid load and inflammatory markers but also reverses the cognitive deficits with memory improvement [97,98].

In human neuronal cells, linagliptin protects against beta-amyloid toxicity and tau hyperphosphorylation, preventing the activation of GSK3β and attenuates intracellular ROS production by stimulating 5’AMP-activated protein kinase (AMPK)-Sirt1 signalling [99]. All these effects contribute to the amelioration of cognitive deficits. Effects of DPP-4i have been investigated in clinical studies. In older patients affected by T2DM and mild cognitive impairment, DPP-4 inhibitor treatment improves glucose control and prevents worsening of cognitive functions [100]. Also, a prospective clinical study evaluating 6-months sitagliptin treatment in elderly diabetic patients reported improvement of cognitive function [118].

**Insulin**

Insulin exerts several effects in the brain regarding cognition, learning, memory and synaptic plasticity, probably involving the complex insulin/IR signalling pathway. Insulin administration attenuates cognitive decline [101,119] and enhances memory in adults with AD [102]. However, insulin systemic administration is associated with reduced penetration in the brain and higher risk of hypoglycaemia. For these reasons, in several clinical studies, intranasal administration of insulin has been tested. After intranasal administration, insulin bypasses the BBB and reaches biologically significant concentrations in the brain [102]. In vitro, insulin inhibits neuronal apoptosis via activation of protein kinase B and in vivo it regulates tau phosphorylation, beta-amyloid precursor protein metabolism and beta-amyloid clearance [103].

Intranasal insulin administration improves memory and enhance mood in healthy adults but also in patients with mild cognitive impairment and late onset AD in which improves cerebral glucose metabolism and preserves volume of brain regions affected by AD pathology [104]. Therapeutic insulin effects on CNS are dose dependent and modulated by APOE genotype, a strong genetic predictor for AD [102].

## 8. Conclusions

It is now recognized that insulin can exert an important effect on brain function. Alterations in insulin metabolism and signalling can contribute to the development of neurodegenerative disorders, such as AD, influencing the regulation of their neuro-pathological hallmarks.

A number of in vivo and in vitro studies confirm the close relationship between T2DM and neurodegeneration and suggest a potential therapeutic role of several anti-diabetics in prevention or treatment of disorders like AD. However, underlying common mechanisms between T2DM and neurodegenerative diseases, as well as determinants of antidiabetic effects on neurodegeneration are not completely known and further studies are needed to clarify these pathophysiological links and the related therapeutic implications.

## Figures and Tables

**Table 1:** Effects of antidiabetic drugs on central nervous system (CNS).

Drugs	Effects on CNS	Ref.
**Metformin**	Restores mitochondria, attenuates AGEs effects through the activation of AMPK in human neural stem cells	[72,73]
	Re-sensitizes impaired insulin signalling and reduces phosphorylation of tau, in neuronal cell lines	[74]
	Induces protein phosphatase 2A and reduces tau phosphorylation in murine neurons of Tau transgenic mouse	[75]
	Attenuates cognitive impairment in obese leptin resistant mice	[76]
	Increases the generation of amyloid beta protein, in human cell models (*negative effect*)	[77]
	Decreases the risk of cognitive decline in diabetic patients	[78]
	Improves depressive and cognitive performance in depressed patients	[79]
	Protective effect on domain of verbal learning, working memory and executive function	[80]
	Increases the risk of cognitive impairment in studies conducted on AD patients (*negative effect*)	[81]
**Sulfonylureas**	Glimepiride protects neurons against beta amyloid induced synapse degeneration, in vitro	[82]
	Gliclazide exerts antioxidant effect in the brain, in diabetic rats	[83]
	Glibenclamide decreases depression and anxiety, in a rat model of AD	[84]
	In combination with metformin, reduce the risk of dementia, in diabetic patients	[85]
**Glitazones**	Neuroprotective effects in AD related to inhibition of inflammation and Aβ deposition	[86]
	Pioglitazone reduces AD-related pathologies suppressing glial activation, in mice	[87]
	Pioglitazone enhances Akt signalling and tau hyperphosphorylation, in mouse model of AD	[88]
	In combination with leptin, pioglitazone reduces brain amyloid levels, in mouse model of AD	[89]
	Pioglitazone improves cognition and regional cerebral blood flow of T2DM patients	[90]
	Pioglitazone may provide an improvement in early stage and in mild to moderate AD in humans	[91]
**GLP-1 RA**	Reduce apoptosis and oxidative stress; ameliorate synaptic plasticity in AD mouse model	[92]
	Influence cellular pathways of neuronal protection and mitochondrial function	[93]
	Reduce tau phosphorylation, prevent synaptic loss, diminish Aβ deposition in AD mouse model	[94,95]
	Prevent the decline of brain glucose metabolism in AD patients with long-standing disease	[96]
**DPP4-i**	Decrease tau phosphorylation, amyloid load and the cognitive deficits with memory improvement	[97,98]
	Improve incretin levels, reduce Aβ deposition, tau phosphorylation, GSK-3β activation and ROS	[99]
	Improve glucose control and prevent worsening of cognitive function, in older patients with T2DM	[100]
**Insulin**	Attenuates cognitive decline and enhances memory in adults with AD	[101,102]
	In vitro inhibits apoptosis; in vivo regulates tau phosphorylation, Aβ metabolism and clearance	[103]
	Improves memory, mood, cerebral glucose metabolism; preserves brain volume in AD patients	[104]
